# Anti-N-Methyl-D-Aspartate Encephalitis as Paraneoplastic Manifestation of Germ-Cells Tumours: A Cases Report and Literature Review

**DOI:** 10.1155/2019/4762937

**Published:** 2019-03-10

**Authors:** Claudia Geraldine Rita, Israel Nieto Gañan, Adriano Jimenez Escrig, Ángela Carrasco Sayalero

**Affiliations:** ^1^Department of Immunology. Hospital Universitario Ramón y Cajal, Ctra. Colmenar Km 9,1, 28034 Madrid, Spain; ^2^Department of Neurology. Hospital Universitario Ramón y Cajal, Ctra. Colmenar Km 9,1, 28034 Madrid, Spain

## Abstract

Anti-N-methyl-D-aspartate (NMDA) receptor encephalitis is the most common form of autoimmune encephalitis, caused by the interaction between an antibody and its target, located on glutamate receptor type N-methyl-D-aspartate (NMDA) of neuronal surface. There is a wide spectrum of clinical features starting by a viral-like prodrome, followed by symptoms such as psychosis, aggressive behaviour, memory loss, seizures, movement disorders, and autonomic instability. Up to 50% of the affected young female patients have germ-cells tumours as ovarian teratoma, making it essential to establish an early diagnosis through detection of specific antibodies in serum and cerebrospinal fluid (CSF). This retrospective observational study was performed in patients whom positive anti-NMDA receptor antibodies have been tested, associated with clinical manifestations that suggest autoimmune encephalitis and a germ-cell tumour confirmed by pathology. Six patients have tested positive for anti-NMDA receptor antibodies associated with a germ-cell tumour and clinical manifestations of autoimmune encephalitis. Management includes aggressive immunosuppression and surgical removal.

## 1. Introduction

Autoimmune encephalitis constitutes a group of neuroinflammatory pathologies, characterized by psychiatric and neurological manifestations caused by the interaction between an antibody (Ab) and its target that can be intracellular or in the cell surface [1, 2]. Anti-N-methyl-D-aspartate (NMDA) antibodies are directed against the NMDA receptor located on neuronal surface, being a heterotetramer composed by two subunits of glutamate ionotropic receptor 1 (GluN1) and two subunits of glutamate ionotropic receptor 2 (GluN2) that acts as a postsynaptic excitatory ionotropic receptor. The GluN1 subunit is mandatory, while the GluN2 subunits (A, B, C, and D) vary depending on the brain region, synaptic or extrasynaptic localization, and brain development. An important aspect of encephalitis mediated by anti-NMDA receptor antibodies is that those directed against the GluN1 subunit are those that result in specific and recognizable syndromes, while those directed against the GluN2 subunits are not associated with any specific syndrome and, in most cases, its clinical and pathogenic value is uncertain [3]. Physiologically, the activation of NMDA receptor facilitates the intracellular increase of ions, initiating a cascade of cell events, which play a crucial role in the process of synaptic plasticity, involved in learning and memory [2].

Anti-NMDA receptor encephalitis is the most common form of autoimmune encephalitis, reaching 1% of all admissions of young adults to an intensive care unit. Up to 50% of the affected young female patients have germ-cells tumours as ovarian teratoma [1]. There is a wide spectrum of clinical features starting by a viral-like prodrome, followed by symptoms such as psychosis, aggressive behaviour, altered mood, insomnia, memory loss, seizures, movement disorders, and pronounced autonomic instability. In addition, respiratory abnormalities sometimes require mechanical ventilation and admission to an intensive care unit [4]. Because of clinical severity, early tumour removal and aggressive immunotherapy are the mainstay of treatment; so it is essential to establish an early diagnosis through detection of specific antibodies in serum and CSF [2, 4]. This study reports six cases of anti-NMDA receptor encephalitis associated with a germ-cell tumour.

## 2. Cases Presentation

We describe cases of six patients with clinical manifestations of autoimmune encephalitis, mediated by anti-NMDA receptor antibodies and a germ-cell tumour confirmed by pathology; the average age of the reported cases was 26-year-old (range 17-33 years), all of them women. Two of the six patients showed dizziness symptom between 1 and 3 months prior to the diagnosis of encephalitis and one had a history of epilepsy during childhood without treatment at current time. One patient had history of cocaine and marijuana abuse, not being referred toxic habits in the rest of the patients.

About clinical features, all patients had anterograde amnesia; four had seizures at any time during the disease; two showed psychiatric symptoms and two patients developed dysautonomy that included one or more of the following: sialorrhea, tachycardia, and lability of blood pressure. Those with movement disorders presented with myoclonus, dyskinesia, and dystonia (Table 1).

**Figure 1:**
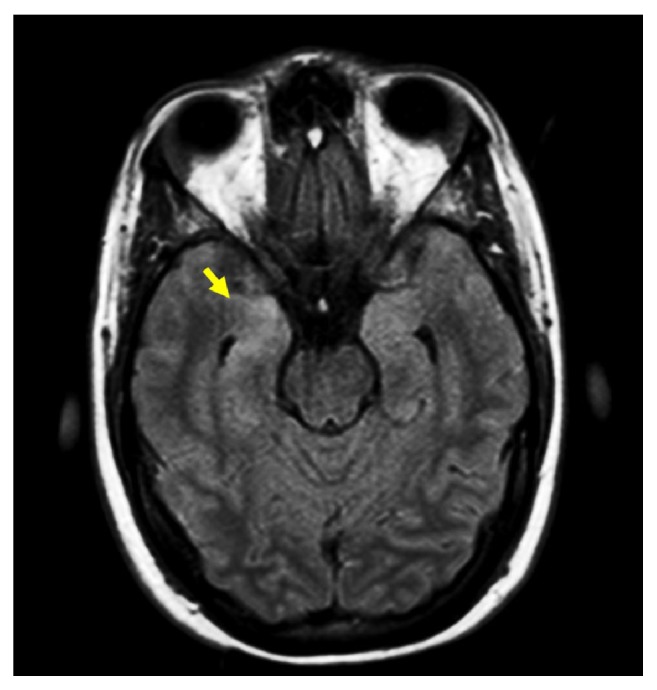
*Brain MRI of patient with positive Anti-NMDAR encephalitis*. Arrow shows cortical hyperintensity in uncus right and hippocampus with T2-FLAIR technique.

**Figure 2:**
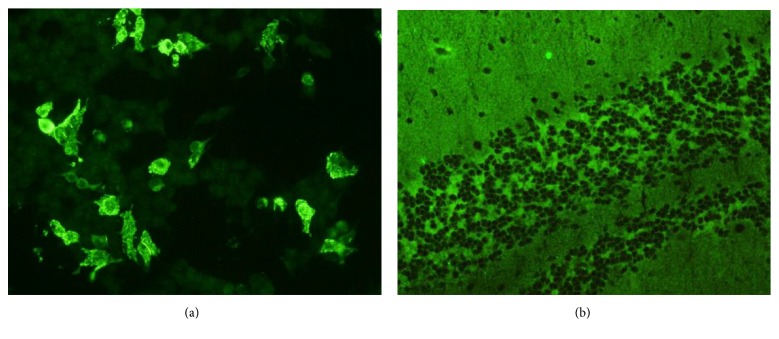
*Anti-NMDAR antibodies detected by indirect immunofluorescence*. (a) Hek293 transfected cells; (b) granular layer of cerebellum in tissue of rat.

The diagnostic approach included blood and CSF tests, radiological images, and electroencephalogram. Pleocytosis in CSF was demonstrated in all cases (range 11-158 cells/mm^3^; median 60.5 cells/mm^3^) and increased protein levels in CSF were observed in two of them (range 0.03-1.16 g/L; median 0.5 g/L); a mirror pattern in oligoclonal bands (OB) was identified in one patient of six. In one case, magnetic resonance imaging (MRI) showed cortical hyperintensity on uncus and right hippocampus suggestive of limbic encephalitis (Figure 1). There was evidence of generalized slowing in the electroencephalogram (EEG) profile in all of them. Anti-NMDA receptor antibodies were determined by indirect immunofluorescence (IIF) in Hek293 transfected cells and rat tissues of cerebellum and hippocampus fixed with acetone in plates, included in the IIFT Glutamate Receptor Mosaic 3 kit of Euroimmun. CSF and serum samples were analyzed without dilution and with a 1:10 dilution, respectively (Figure 2). In two cases detection was performed twice to confirm the results. All patients had extensive serum and CSF diagnostic tests with negative or normal results for viral or bacterial infections; autoimmunity screening was performed in six patients with positive ANA in two cases (Table 1).

Computed tomography or ultrasound demonstrated an ovarian mass in all patients. Complete tumour resection was done in all cases and pathological studies showed mature ovarian cystic teratoma in five of them and one case had stage IA immature teratoma. Tumour was unilateral in five cases and bilateral in one; CA 125 was measured in all patients, being above reference range in all of them, with a maximum of 44 IU/ml in the patient who presented immature teratoma.

**Figure 3:**
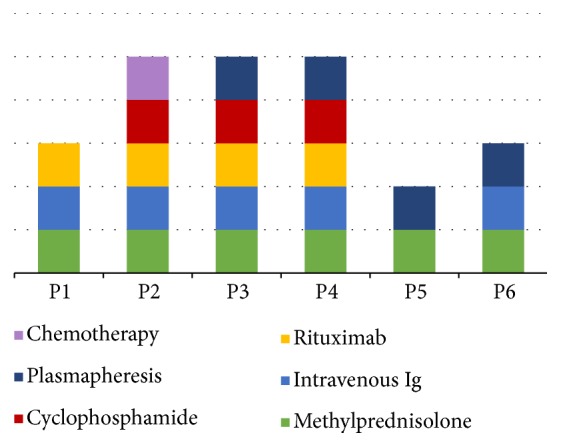
*Treatment grouped by patient*. P1, P3, P4, and P5 received methylprednisolone (1 g IV daily) during 5 days; P2 and P6 received methylprednisolone according their weight (1mg/kg) also during 5 days. IVIG was administered in five cases at immunomodulatory doses (400-800mg/kg/day). P2 and P3 needed cyclophosphamide (750 mg/m^2^ IV) every three weeks during 4 cycles; P4 received cyclophosphamide (750 mg/m^2^ IV) 2 cycles with 6 weeks between each one. Rituximab was administered every two weeks (375 mg/m^2^ IV) for 2 cycles in P1, P2, P3, and P4. In P2 it was necessary to add four cycles of chemotherapy for immature teratoma repeated every 21 days with bleomycin (30 IU IV every week), etoposide (100mg/m2 IV daily for 5 days), and cisplatin (20mg/m2 IV daily for 5 days).

Patients were treated with immunomodulatory therapy according to the severity of their case. Overall, all of them received corticosteroid therapy, four with boluses of methylprednisolone, 1 g intravenous during 5 days and two cases received methylprednisolone according their weight by 1mg/kg also during 5 days; up to 50% needed a cycle of intravenous immunoglobulins (IVIG) at immunomodulatory doses (400-800mg/kg/day) during 5 or 6 days and one of them needed a second cycle of IVIG; four patients received rituximab according to their body surface area with a 375 mg/m^2^ IV infusion every two weeks for 2 cycles; four patients needed plasma exchange and three cyclophosphamide 750 mg/m^2^ IV; one case received 2 cycles with 6 weeks between each one and two patients received 4 cycles every three weeks. In the case of immature teratoma it was necessary to add four cycles of chemotherapy repeated every 21 days with bleomycin (30 IU IV every week, for three doses), etoposide (100mg/m2 IV daily for 5 days), and cisplatin (20mg/m2 IV daily for 5 days) (Figure 3). After treatment, patients showed gradual improvement in their symptoms over months.

## 3. Discussion

N-methyl-D-aspartate receptor channel is a tetramer composed by two subunits of GluN1 and two subunits of GluN2; in 2007 Dalmau et al. described for the first time the anti-NMDA receptor antibody associated with 12 cases of encephalitis [5]. This antibody is against the GluN1 subunit and it is present in neuronal surface of the hippocampus, cerebral cortex, basal ganglia, and thalamus [2, 6]. Hughes et al. suggested that GluN1 antibodies from patients with anti-NMDA receptor encephalitis decrease glutamatergic synaptic function, which may underlie the deficits of memory, behaviour, and cognition that are hallmarks of anti-NMDA receptor encephalitis [6]. Currently it is a very common cause of encephalitis reaching 1% of all admissions of young adults to an intensive care unit [1].

According to clinical description, patients with NMDA receptor encephalitis show a recognizable syndrome, starting with a rapidly progressive viral-like symptoms such as headache and hyperthermia, which progresses in days to more complex clinical features including psychosis, delusions, hallucinations, agitation, aggression, catatonia, insomnia, speech dysfunction follow due to dyskinesias, memory deficits, autonomic instability, seizures, and even decreased level of consciousness [3, 4, 7, 8]. These unspecific clinical features are often mistaken as viral encephalitis, primary psychiatric disorders, drug abuse, or neuroleptic malignant syndrome, which could delay diagnosis and treatment [3].

Clinical features observed in present study were similar to previous reports [1, 4, 5, 7, 8]. Memory deficit is commonly described in this disorder; however, it might be undervalued because of difficulties to assess it in young patients or patients with psychiatric manifestations [4]; in the present study 100% of cases had anterograde amnesia before other encephalitis symptoms appeared. According to Gresa-Arribas et al., one of the first neurological symptoms observed in young patients with anti-NMDA receptor encephalitis are generalized (72%) and focal seizure (42%) [9]; in our series up to 50% of cases presented with seizures at any time during course of the disease; two debuted with generalized tonic-clonic seizures and one case presented with complex partial crisis. Kaneko et al. described movement disorder in all patients studied [10]; in present study there was one case of dystonia, one case of myoclonus, and one case of dyskinesia. Autonomic dysfunction has been previously reported in anti-NMDA receptor encephalitis [1, 3, 4, 7, 11]. In a series of 100 patients with anti-NMDA receptor encephalitis, 37 patients presented with cardiac arrhythmias [7] and there are at least 2 reported cases who required a permanent pacemaker to control cardiac arrhythmia [11]. It has been hypothesized that autonomic instability might be associated with seizure activity or induced by sympathetic and parasympathetic stimuli [11]. In our series, 2 patients presented with dysautonomy; one of them had lability of blood pressure, and the other one tachycardia and sialorrhea who received local botulinum toxin injections in two occasions to be controlled. Teenagers and young adults usually showed abnormal behaviour (psychosis, delusions, hallucinations, agitation, aggression, or catatonia) [4, 7, 8]. This review found two patients with psychiatric symptoms, such as auditory hallucinations and delusion, requiring antipsychotics.

The diagnosis of anti-NMDA receptor encephalitis is confirmed by the detection of serum or CSF antibodies against the GluN1 subunit of the NMDA receptor [1–3]. A significant aspect of encephalitis mediated by anti-NMDA receptor antibodies is that those directed against the GluN1 subunit are those that result in specific and recognizable syndromes, while those directed against the GluN2 subunits are not associated with any specific syndrome [3]. Dalmau et al. considered that serum testing is less reliable, with false negative results in up to 14% of cases [1]; on the other hand Guasp et al. suggested that there is an increasing number of patients mistakenly diagnosed due to false positive serum results recommending confirmation by CSF examination [3]. By contrast Irani et al. reported that serum levels of anti-NMDA receptor antibodies were similar or higher to those of CSF [12]. In this study determinations of anti-NMDA receptor were performed by IIF in serum and CSF and were concordant in 100% of them (Figure 2).

Diagnosis approach also included MRI and EEG; in some cases MRI scan was normal or showed unilateral changes [1]. In a study of 100 patients with anti-NMDA-receptor encephalitis only 55% of patients had increased T2-FLAIR signal in one or several brain regions, without significant correlation with patients' symptoms [7]. In present series only one case shows cortical hyperintensity on uncus of right hippocampus (Figure 1). EEG profile was abnormal in most patients, usually showing nonspecific, slow, and disorganized activity. All cases reported in this study had a generalized slow tracing in EEG. Another analysis of CSF usually shows moderate lymphocytic pleocytosis with normal or mildly increased protein concentration [5, 7]; all cases in this study had pleocytosis in CSF. There was evidence that more than 50% of patients showed specific oligoclonal bands [7]; nevertheless in present series it was one case of mirror pattern on OB; mirror OB have been reported in patients with autoantibody-associated disorders, such as NMDA receptor encephalitis and VGKC encephalopathy. This pattern implies the presence of clonal IgG in both CSF and serum and it means that the production of autoantibody might be first triggered in the periphery, rather than in the CNS so they may be an important biomarker in inflammatory central nervous system disorders [13, 14].

In this study 17 percent of the patients with a positive NMDA receptor antibody had an ovarian teratoma, which is significantly lower than in other published series; some epidemiological studies suggested that this association depends on demographic features of the population analyzed [15]. Case reports and observational studies have estimated that 36%-50% of patients with anti-NMDA receptor encephalitis have an ovarian teratoma [16, 17]. Gresa-Arribas et al. reported more frequently NMDA receptor antibodies in patients who had an underlying teratoma than in those who did not have a tumour [9]. A mature cystic ovarian teratoma was identified in 5 patients in this study and one patient had an immature teratoma confirmed by pathology; in a cohort of 252 patients an immature ovarian teratoma was diagnosed in 11% of them [18]. One case of bilateral teratoma was observed in this study with similar outcome compared to other patients with unilateral tumour; Lee et al. reported a case of fulminant course in a patient with bilateral teratoma suggesting that it might be related to higher antibody titers [19].

The optimal management of anti-NMDA receptor encephalitis includes immunotherapy and removal of the immunological trigger, such as teratoma or another tumour [1, 5]. First-line immunosuppressive therapy includes methylprednisolone 1 g per day for 5 days and concomitant intravenous immunoglobulins (0.4 g/kg per day for 5 days) or plasma exchange. Second-line therapy consists of rituximab (375 mg m^2^ 1 week for 4 weeks) combined with cyclophosphamide (750 mg m^2^) given with the first dose of rituximab, followed by monthly cycles of cyclophosphamide [15]. In present study 2 patients showed improvement of the neurological outcome after tumour resection and first-line therapy, alone or combined, while 4 patients need a second-line immunotherapy (Figure 3). In a study of 577 patients the combination of first-line immunotherapy more frequently used was steroids and IVIG [8], similar to combination observed in present series. Dalmau et al. reported that 80% of patients with a tumour (mostly teratomas) had substantial improvement after tumour removal and first-line immunotherapy [15]. These observations and the possible rapid neurological improvement after tumour resection suggest that a peripheral immune response against tumour autoantigens could be involved in the production of anti-NMDA receptor autoantibodies [18].

Recovery from this disorder is typically slow, and symptoms may relapse, especially in patients with undetected or recurrent tumours [7]. In accordance with Titularer et al. 53% of patients had clinical improvement within 4 weeks, and 81% had substantial recovery (i.e., mild or no residual symptoms) at 24 months [8]. Patients analyzed in this study had no relapses reported.

## 4. Conclusion

Anti-NMDA receptor encephalitis is a multifaceted disease associated with serious complications, which may require an early diagnosis and extensive studies to establish the presence of an underlying germ-cell tumour to perform a prompt surgery and aggressive immunotherapy.

## Figures and Tables

**Table 1:** Clinical and immunological features of patients with Anti-NMDAR encephalitis.

ID	Sex, Age (y/o)	Clinical features	CSF	1st brain MRI	Anti- NMDAR	Autoimmunity	Tumor pathology
P1	F, 29	Anterograde amnesia	Cell: 13	Normal	(+) CSF and serum	ANA 1/160	Mature ovarian cystic teratoma
		Myoclonias	Prot: 0.48			ENAS (-)	
		Auditory hallucinations	OB: Negative				

P2	F, 27	Anterograde amnesia Tonic-clonic seizure	Cell: 15	Normal	(+) CSF and serum	ANA 1/640	Immature Teratoma Stage IA
			Prot: 0.03			ENAS (+)	
			OB: Negative			Anti Ro (+)	

P3	F, 33	Anterograde amnesia	Cell: 159	Cortical hyperintensity in uncus and hippocampus	(+) CSF and serum	Negative	Mature ovarian cystic teratoma
		Dyskinesia	Prot: 1.16				
		Sialorrhea and tachycardia	OB: mirror pattern				
		Tonic seizure					

P4	F, 27	Anterograde amnesia	Cell: 158	Normal	(+) CSF and serum	Negative	Mature ovarian cystic teratoma
		Tonic seizure	Prot: 1.03				
		Delusions	OB: Negative				

P5	F, 24	Anterograde amnesia	Cell: 11	Normal	(+) CSF and serum	Negative	Mature ovarian teratoma
		Lability of blood pressure	Prot: 0.42				
		Complex partial seizures	OB: Negative				

P6	F, 17	Anterograde amnesia Dystonias	Cell: 17	Normal	(+) CSF and serum	Negative	Bilateral mature ovarian cystic teratoma
			Prot: 0.21				
			OB: Negative				

Cell: cells/mm^3^; Prot: g/L.

## Abbreviations

ANA: Antinuclear antibodies
CSF: Cerebrospinal fluid
EEG: Electroencephalogram
GluN1: Glutamate ionotropic receptor subunit 1
GluN2: Glutamate ionotropic receptor subunit 2
IIF: Indirect immunofluorescence
IVIG: Intravenous immunoglobulins
MRI: Magnetic resonance imaging
NMDA: Anti-N-methyl-D-aspartate
OB: Oligoclonal bands
T2-FLAIR: T2-weighted-Fluid-Attenuated Inversion Recovery.
